# Mass Photometry Reveals Distinct ACE2 Binding Stoichiometries across SARS-CoV‑2 Omicron Subvariants

**DOI:** 10.1021/acs.jpcb.6c00027

**Published:** 2026-04-30

**Authors:** Wei-Cheng Hsiao, Tsung-Sheng Chiang, Yu-Xi Tsai, Min-Feng Hsu, Shang-Te Danny Hsu

**Affiliations:** 1 Institute of Biological Chemistry, 38017 Academia Sinica, Taipei 11529, Taiwan; 2 Institute of Biochemical Sciences, National Taiwan University, Taipei 10617, Taiwan; 3 International Institute for Sustainability with Knotted Chiral Meta Matter (WPI-SKCM^2^), Hiroshima University, 1-3-1 Kagamiyama, Higashi-Hiroshima, Hiroshima739-8531, Japan; 4 Department of Biochemistry, Microbiology, and Immunology, Faculty of Medicine, University of Ottawa, Ottawa, Ontario K1H 8M5, Canada

## Abstract

Since late 2021, the SARS-CoV-2 Omicron variant has rapidly accumulated mutations in its spike (S) protein, leading to increased transmissibility and immune evasion. The COVID-19 pandemic caused by SARS-CoV-2 infections poses a significant global health challenge. While the binding affinity of Omicron S proteins to host receptor ACE2 has been extensively characterized, the binding stoichiometry across subvariants remains unclear. We used mass photometry (MP) to determine the ACE2 binding stoichiometry to different Omicron S subvariants. MP revealed diverse stoichiometries across subvariants, indicating that S protein mutations modulate ACE2 engagement beyond mere affinity. These findings reveal a nonlinear evolutionary trajectory of ACE2 engagement among Omicron subvariants, underscoring stoichiometry as a key variable in viral adaptation.

## Graphical abstract

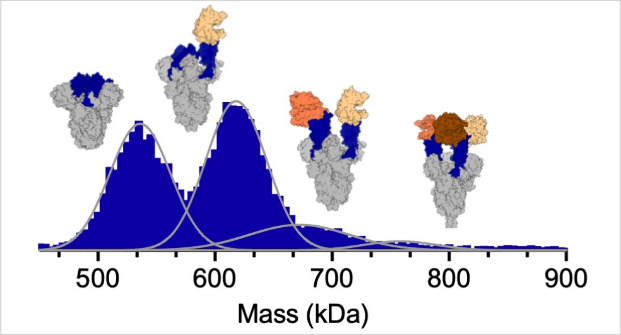

## Introduction

Since late 2021, the Omicron variants of SARS-CoV-2 have continued to evolve among human populations, causing waves of infection cases. The emergence of the subvariants from BA.1, BA.2, BA.4/5, BQ.1, XBB, EG.5, to JN.1 remains a significant concern for global public health.^1,2^ Further research is needed to understand how these variants escape host immunity while maintaining high-affinity binding to the host receptor, angiotensin-converting enzyme 2 (ACE2). The spike (S) protein of SARS-CoV-2 on the viral surface is a heavily glycosylated trimeric complex, responsible for mediating viral entry into host cells by binding to ACE2 through its receptor binding domain (RBD).^3^ The RBD must undergo conformational changes from the downward closed conformation to the upward open conformation to bind to ACE2. Understanding the nuances of this interaction is crucial for predicting future evolutionary trajectories and developing broadly effective countermeasures.

Despite extensive quantitative characterizations of ACE2 binding affinities for various Omicron S protein variants using biophysical techniques such as surface plasmon resonance (SPR) and biolayer interferometry (BLI),^4,5^ a critical but less explored aspect of SARS-CoV-2 S protein:ACE2 interaction is the binding stoichiometry. The SARS-CoV-2 S protein and ACE2 function as a homotrimer and a homodimer, respectively; the binding stoichiometry between the two could modulate avidity, receptor clustering on the cell surface, and the efficiency of subsequent membrane fusion events leading to viral entry. Binding stoichiometry can be best determined by isothermal titration calorimetry (ITC).^6^ However, ITC requires a large quantity of proteins that may not be tractable for the SARS-CoV-2 S protein and ACE2. Moreover, SARS-CoV-2 S protein binding to ACE2 does not follow a single binding stoichiometry but rather a distribution of binding modes, making it challenging for ITC data analyses, which require a single binding model for fitting. Native mass spectrometry can provide insights into the complex formation in the gas phase.^7–9^ However, the inherent complexity of glycosylated proteins can complicate spectral interpretation and quantification. Native mass spectrometry also requires specialized instrumentation that may not be easily accessible to researchers.

By contrast, mass photometry (MP) is an emerging label-free technique that enables direct measurements of the molecular masses of individual particles in solution. MP offers distinct advantages for studying S -ACE2 interactions: First, MP requires minimal sample consumption, typically on the pico to nanomole scale; second, MP can resolve complex assemblies over a broad mass distribution range, allowing quantitation of the relative populations of different stoichiometries; third, MP offers direct assessment of molecular mass under near-native, solution-based conditions, preserving physiologically relevant interactions; finally, MP data acquisitions can be completed in minutes, thus enabling high-throughput analyses of multiple variants.^10,11^ In this study, we leveraged the power of MP to assess the ACE2 binding stoichiometries of a panel of SARS-CoV-2 Omicron subvariants. Our findings offered new insights into how different Omicron variants may engage with ACE2 in the context of binding stoichiometry, fulfilling a knowledge gap in our understanding of how SARS-CoV-2 variants evolve under selection pressures.

## Material and Method

### Protein Plasmid Preparation, Expression, and Purification

S proteins of SARS-CoV-2 Omicron subvariants (BA.1, BA.2, BA.2.75, BA.4/5, BQ.1, EG.5.1, XBB.1.5, JN.1) with hexaproline and furin cleavage site mutations were expressed from pcDNA3.1 vectors in Expi293 cells (Gibco, USA) and harvested after 5 days. Clarified supernatants were purified via Ni-NTA resin (Roche, Germany) using a PD-10 column (Cytiva, USA), washed with 15 column volume (CV) of Wash A (20 mM Tris-HCl (pH 7.6), 300 mM NaCl, 10 mM imidazole), and eluted with 10 CV of Elution Buffer (20 mM Tris-HCl (pH 7.6), 150 mM NaCl, 300 mM imidazole), followed by size-exclusion chromatography (SEC) using a Superose 6 Increase 10/300 column (Cytiva, USA) with phosphate buffer saline (PBS; NaCl: 137 mM, KCl: 2.7 mM, Na_2_HPO_4_: 10 mM, KH_2_PO_4_: 1.8 mM) as the mobile phase.

The DNA sequence corresponding to human ACE2 (residues 1–615, extracellular domain) with a C-terminal octa-histidine (His_8_) tag was cloned into a pcDNA3 plasmid, and delivered into Expi293 cells (Gibco, USA) for transient expression, which was harvested after 4 days. Clarified supernatant was purified via Ni-NTA resin (Roche, Germany) using a PD-10 column (Cytiva, USA). The column was washed sequentially with 15 CV of: Wash A; Wash B (20 mM Tris-HCl (pH 7.6), 500 mM NaCl, 15 mM imidazole); then Wash A again, before eluting with 10 CV of Elution Buffer, followed by SEC on a Superdex 200 Increase 10/300 column (Cytiva, USA) with 50 mM sodium phosphate, pH 6.0.

The ectodomain of human ACE2 was fused to a human IgG Fc domain and cloned into the pcDNA3.4 vector for expression in Expi293F cells. The secreted hACE2-Fc was harvested by centrifugation (8,000 rpm, 15 min, 8 °C) and filtered. The supernatant was adjusted to 20 mM Tris-HCl (pH 8.0) and captured using a HiTrap Q FF anion exchange column (5 mL, Cytiva, USA) on an ÄKTA Pure M FPLC system (Cytiva, USA). The protein was eluted with a linear gradient of 1 M NaCl. Pooled fractions were concentrated via a 100 kDa MWCO centrifugal filter and further refined by SEC using a Superose 6 Increase 10/300 GL column (Cytiva, USA) equilibrated in PBS (pH 7.4). Fractions corresponding to the dimeric form were collected and used to perform biolayer interferometry (BLI) experiments.

### Mass Photometry (MP) Measurement

MP measurements were performed using the Refeyn TwoMP system (Refeyn, UK). Glass coverslips were cleaned by sequentially rinsing them with Milli-Q water and isopropanol, followed by drying under a nitrogen stream. The cleaned coverslips were assembled for sample delivery using silicone gaskets (6 × 3 mm diameter × 1 mm depth, Refeyn, UK). Before sample measurements, the coverslips were placed on the MP sample stage, and a single well of the gasket was filled with 16–18 μL of PBS to establish focus and ensure a low background signal-to-noise ratio. Mass calibration was performed using BSA (monomeric form, 66 kDa and dimeric form, 132 kDa) and bovine thyroglobulin (TG, 670 kDa). S variants and ACE2 were mixed with different molar ratios ranging from 1:1. 1:2, 1:4 to 1:8, with a final concentration of 5 nM for S variants and 5–40 nM for ACE2. The S-ACE2 mixtures were incubated at room temperature at final concentrations of 25 nM and 25–200 nM, respectively, for 1 h, followed by a five-fold dilution immediately before the MP measurements.

### Bio-Layer Interferometry (BLI)

Binding kinetics of SARS-CoV-2 S protein variants to human ACE2-Fc (hACE2-Fc) were measured using a Gator Plus system (Gator Bio, USA). Anti-human Fc capture (AHC) biosensors were hydrated for 30 min in BLI assay buffer (PBS (pH 7.4), 0.1% BSA, 0.02% Tween-20) prior to use. Sensors were loaded with hACE2-Fc (ligand) to a target shift of 0.35 nm and stabilized for 120 s in assay buffer to establish a baseline.

For kinetic analysis, loaded sensors were immersed in a two-fold serial dilution of trimeric S variants (analyte), ranging from 50 nM to 1.563 nM. The association phase was monitored for 300 s, followed by a dissociation phase in assay buffer for 300 s. To account for nonspecific binding and baseline drift, data were double-referenced using both an unloaded reference sensor and a buffer-only control. Data processing and global fitting were performed using GatorOne software (Gator Bio, USA). Sensorgrams were fitted to a 1:1 binding model to calculate the association rate constant (*k*_off_), dissociation rate constant (*k*_on_), and the equilibrium dissociation constant (*K*_D_).

### GlycoSHIELD Modeling of the JN.1 S Protein

The fully glycosylated atomic model of the JN.1 S protein was constructed using the GlycoSHIELD pipeline^12^ in coarse-grained modeling mode with a 3.5 Å distance threshold. All putative N-glycosylation sites were assigned specific glycan determined by MS;^13^ detail glycan classifications and their corresponding sit assignments are provided in Table S1, which were grafted from the GlycoSHIELD glycan trajectory library onto the static S protein structure. A corresponding membrane environment was generated using the CHARM-GUI Membrane Builder^14,15^ to provide a realistic structural context. The resulting GlycoSHIELD model was visualized and rendered by ChimeraX^16^ to illustrate the shielding effects of glycosylation.

### RBD-up Angle Calculation of Currently Available Cryo-EM Structures of SARS-CoV-2 S Protein on Protein Data Bank (PDB)

The SARS-CoV-2 S protein structures from the Protein Data Bank (PDB)^17^ were classified into three groups: apo form, ACE2-bound form, and antibody-bound form. The RBD tilting angles for the individual photometers were calculated using an in-house Python pipeline. For each structure, the coordinates of the starting residues (residue 319) from the three RBDs were extracted by PyMOL^18^ using the “get_coords” and “centerofmass” commands. The centers of mass (COMs) of the three RBDs were computed to define reference points for each protomer. Vectors from each COM to its corresponding starting residue were generated, and the cross product of two vectors relative to the third defined the normal vector representing the RBD plane. The angles between this normal and each chain-specific RBD vector were measured to quantify the angle.

## Results

To investigate the evolution of ACE2 engagement by the Omicron S protein variants, we generated a library of engineered SARS-CoV-2 S protein ectodomain variants corresponding to the Omicron subvariants, BA.1, BA.2, BA.4/5, BA.2.75, BQ.1, XBB.1.5, EG.5.1, and JN.1. These constructs harbored the HexaPro substitutions,^19^ mutations in the polybasic furin cleavage loop^20^ and a C-terminal helical bundle foldon^21^ to stabilize the prefusion trimeric assembly. The purity of the recombinant S variants and ACE2 was confirmed by SDS-PAGE (Figure S1) and the trimeric assemblies of S variants were confirmed by MP which revealed monodispersed peaks with apparent molecular weights ranging from 530 to 550 kDa (Figure S2), with slight variations likely attributable to differing glycosylation levels.

To assess the binding stoichiometry between S variants and ACE2 as a function of S:ACE2 mixing ratio, we first performed MP analyses by preincubating S variants with ACE2 in different molar ratios, namely 1:1, 1:2, 1:4, and 1:8. Immediately before MP measurements, the S variants and ACE2 mixtures were diluted to nM range close to the reported binding affinities (5–10 nM)^4,5^ (see Materials and Methods) to monitor the relative populations of apo S, S:ACE2, S:ACE2_2_ and S:ACE2_3_ as a function of mixing ratios (Figure 1 and Figure S2). The resulting mass distribution histograms observed in MP measurements were fit to the sum of multiple Gaussian distributions, corresponding to four distinct species: the unbound S trimer (S; 530–550 kDa), the S trimer bound to one ACE2 (S:ACE2), two ACE2 (S:ACE2_2_), or three ACE2 (S:ACE2_3_) with an increment of ca. 85 kDa (Figure S2). The relative population of each species provides a direct measure of the distribution of different binding stoichiometries for each variant in aqueous solution (Figure 1). In general, ACE2-bound populations with different binding stoichiometries increased on increasing molar ratio of ACE2 with respect to S variants. The population shifts started to level off at 1:4 mixing ratio, which exhibited the optimal dynamic range between different populations for comparative analyses. We, therefore, opted for the 1:4 mixing ratio for subsequent analyses in technical triplicates.

**Figure 1:**
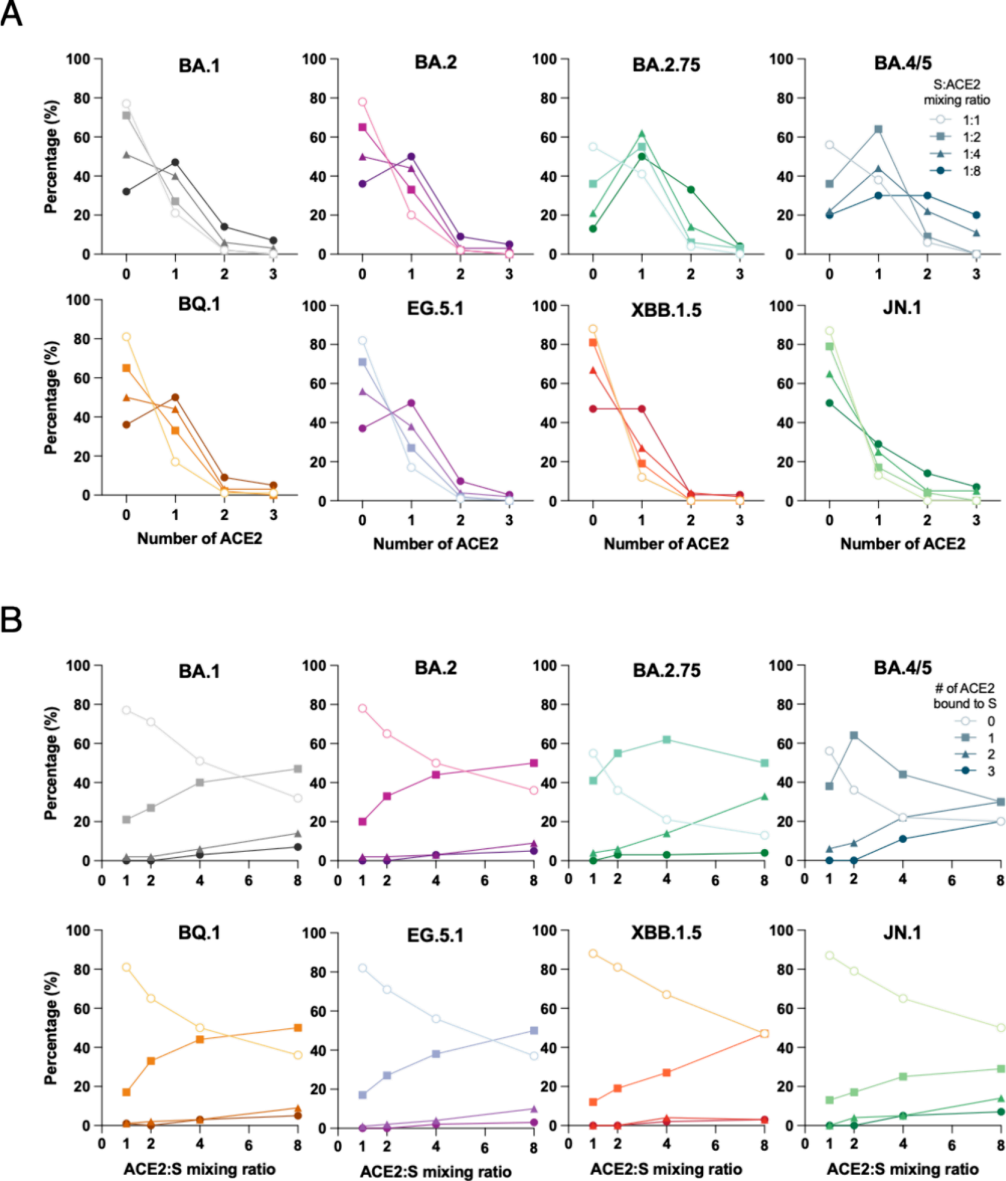
Stoichiometric distributions of ACE2 binding across different SARS-CoV-2 Omicron subvariants. (A) The percentage of S proteins bound to 0, 1, 2, or 3 ACE2 is shown for the eight Omicron subvariants (BA.1, BA.2, BA.2.75, BA.4/5, BQ.1, EG.5.1, XBB.1.5, and JN.1 as indicated above individual panels). The data points derived from S:ACE2 premixing ratios of 1:1, 1:2, 1:4 and 1:8 are shown in open circles, filled squares, filled triangles and filled circles, respectively. (B) Relative populations of different ACE2 binding stoichiometries in percentage as a function of ACE2:S mixing ratio. The data points corresponding to 0, 1, 2, and 3 ACE2-bound S variants are shown in open circles, filled squares, filled triangles, and filled circles, respectively.

We carried out MP measurements in technical triplicate with a 1:4 molar of preincubated S:ACE2 mixture to derive the relative populations of free S, S:ACE2, S:ACE2_2_, and S:ACE2_3_ (Figure 2, Figure S3, and Table 1). Comparison of the relative populations of the individual ACE2 binding stoichiometries of different Omicron subvariants revealed an intriguing evolutionary path for ACE2 engagements. For the early Omicron variants – BA.1 and BA.2 – about 50% of their S proteins existed in an 1:1 binding stoichiometry, i.e., S:ACE2, while about 10% and 3% existed in 1:2 (S:ACE2_2_) and 1:3 (S:ACE2_3_) binding stoichiometries, respectively. About 38% of the overall population was not involved in ACE2 binding (Figure 2). The following BA.4/5 variant exhibited the highest populations of S:ACE2_2_ (24%) and S:ACE2_3_ (10%), followed by the BA2.75 variant that exhibited the highest population of S:ACE2 (62%), the second largest population of S:ACE2_2_ (15%) and the smallest apo S population (20%). After BA.2.75, a reversing trend of ACE2 binding stoichiometry was observed for the ″second-generation″ Omicron variants. BQ.1 exhibited an increased apo form population (36%), accompanied by reduced populations of S:ACE2 (46%) and the second largest population of S:ACE2_2_ (11%). Further reduction of the S:ACE2_2_ population (7%) was observed for the subsequent XBB.1.5 subvariant, accompanied by an increase of the S:ACE2 population (51%). For the more recent Omicron subvariants – EG.5.1 and JN.1 – both exhibited the highest free S populations (58%) with significantly reduced S:ACE2 populations (36% and 29%, respectively). Although JN.1. exhibited a slightly increased S:ACE2_2_ population (9%), the overall trend after the BQ.1 subvariant was an increase in the free S populations. Only ca. 40% of the S variants were engaged with ACE2 binding, primarily in a 1:1 binding stoichiometry, i.e., S:ACE2.

**Figure 2:**
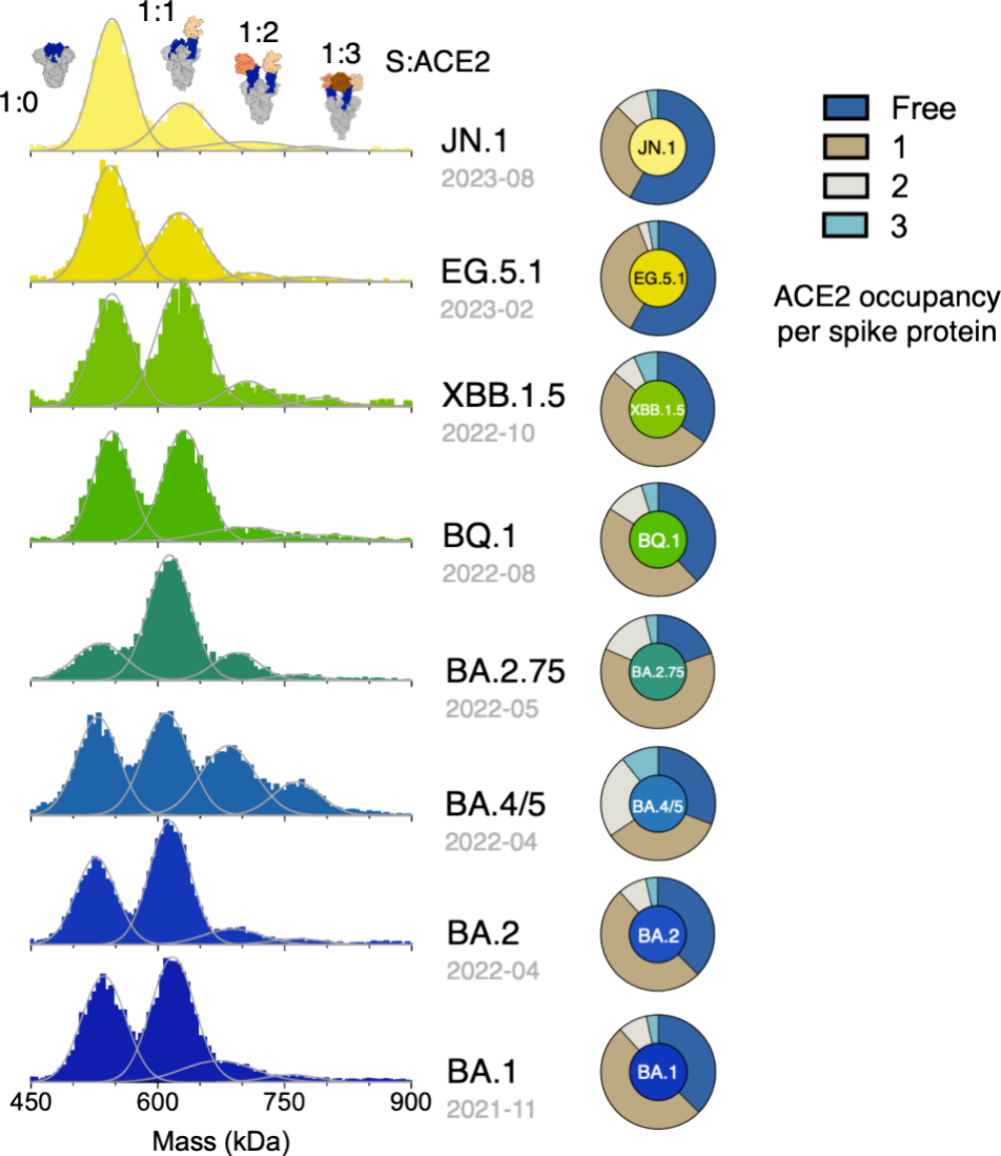
MP analysis of binding stoichiometry between SARS-CoV-2 Omicron S variants and ACE2 in chronological order. Normalized mass distributions for mixtures of ACE2 monomer and various S variants (BA.1, BA.2, BA.4/5, BA.2.75, BQ.1, XBB.1.5, EG.5.1, and JN.1) premixed at a 1:4 molar ratio (5 nM S protein: 20 nM ACE2), presented in chronological order of their emergence (indicated by gray fonts below their subvariant names). The solid lines represent the fitted Gaussian distributions. Pie charts quantifying the relative populations of different binding stoichiometries (free S, S:ACE2, S:ACE2_2_, S:ACE2_3_, and S:ACE2 complexes as schematically illustrated on top) derived from the MP data.

**Table 1:** Distributions of Distinct S:ACE2 Binding Stoichiometries across Different Omicron Subvariants^a^

ACE2 binding stoichiometry	BA.1	BA.2	BA.4/5	BA.2.75	BQ.1	XBB.1.5	EG.5.1	JN.1
free	38.0 ± 2.0	37.5 ± 4.3	30.9 ± 2.3	19.7 ± 2.4	38.1 ± 1.3	34.7 ± 3.1	58.0 ± 2.7	58.1 ± 3.2
S:ACE2	46.7 ± 1.6	50.9 ± 1.7	34.6 ± 1.8	61.8 ± 1.9	46.0 ± 0.3	51.4 ± 4.4	36.2 ± 3.7	29.3 ± 1.0
S:ACE2_2_	12.1 ± 0.7	8.2 ± 2.0	23.8 ± 2.4	15.0 ± 2.3	11.1 ± 1.0	7.0 ± 0.6	2.9 ± 0.5	9.5 ± 1.5
S:ACE2_3_	3.2 ± 1.0	3.4 ± 1.1	10.7 ± 1.8	3.5 ± 1.0	4.8 ± 0.4	6.9 ± 0.7	2.9 ± 0.5	3.1 ± 1.0

a The relative populations (in percentage) of S variants bound to 0, 1, 2, and 3 ACE2, designated as free, S:ACE2, S:ACE2_2_, and S:ACE2_3_, respectively. The values represent the mean ± standard deviation (SD) derived from technical triplicates.

Throughout the brief but rapid evolution of SARS-CoV-2, a drastic jump in the number of mutations in the S protein was observed since the emergence of the Omicron variants (Figure 3A). The RBD progressively accumulates mutations (from 15 mutations in BA.1 to 23 mutations in EG.5.1) until another jump in the JN.1 variant (28 mutations in the RBD and 63 mutations in the entire S protein; Figure 3A). Importantly, two new N-linked glycosylation sites (N245 and N354) near the ACE2 binding interface (magenta ensembles in Figure 3B) are only found in the JN.1 variant.

**Figure 3:**
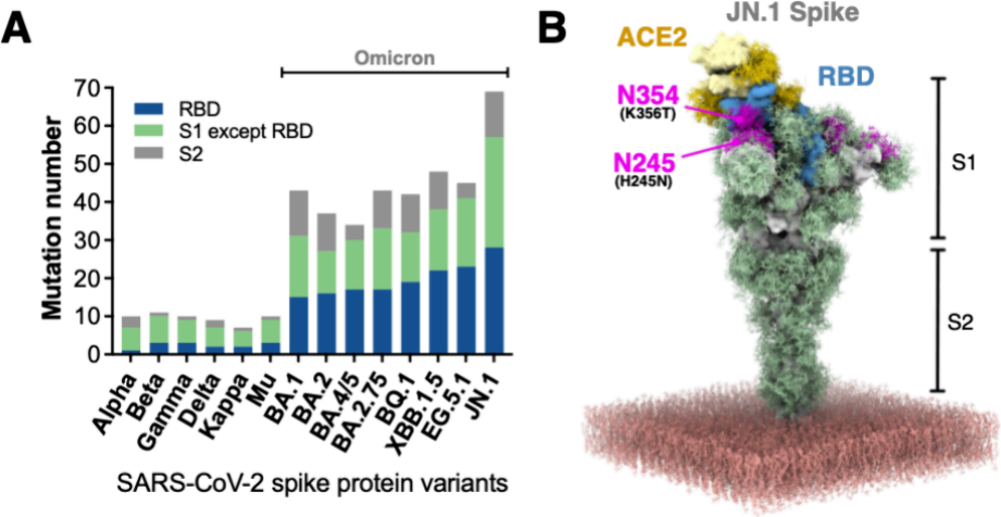
S protein mutation landscape and JN.1-specific glycosylation. (A) Bar chart quantifying S protein mutations across SARS-CoV-2 variants (Alpha to recent Omicron subvariants). Mutations are categorized by protein segments: RBD domain (blue), S1 subunit excluding RBD (green), and S2 subunit (gray). (B) GlycoSHIELD model of the JN.1 S protein illustrating the glycan shield (green) on the protein surface. Novel glycosylation sites specific to JN.1 (N245 and N354) are highlighted in magenta, positioned near the RBD (cyan) and ACE2 (yellow). The atomic model is generated by grafting presimulated glycan conformation ensembles onto the specified N-glycosylation sites through exclusion of conformers that are sterically clashed with the input structure. The protein structure is static, not a molecular dynamics simulation generated model.

To further characterize the interactions between the Omicron S variants and ACE2, we analyzed the binding kinetics by BLI using an hACE2-Fc fusion construct, which self-dimerizes through the Fc domain. This bivalent format mimics the native dimeric state of ACE2 ^22^ and accounts for the avidity effects inherent to the virus-host interface. All tested subvariants exhibited high-avidity binding, reaching a high-affinity plateau with apparent dissociation constants of approximately 1 nM (Figure 4). These results demonstrate that while the stoichiometric distributions observed in our MP analyses vary significantly across the evolutionary trajectory, all Omicron S variants exhibited strong binding affinity toward ACE2 in the low nM range. Furthermore, there is no apparent correlation between the BLI-derived binding affinities with the MP-dirived ACE2 binding stoichiometry. The two analytical tools, therefore, provide complementary information about the S:ACE2 interactions.

**Figure 4:**
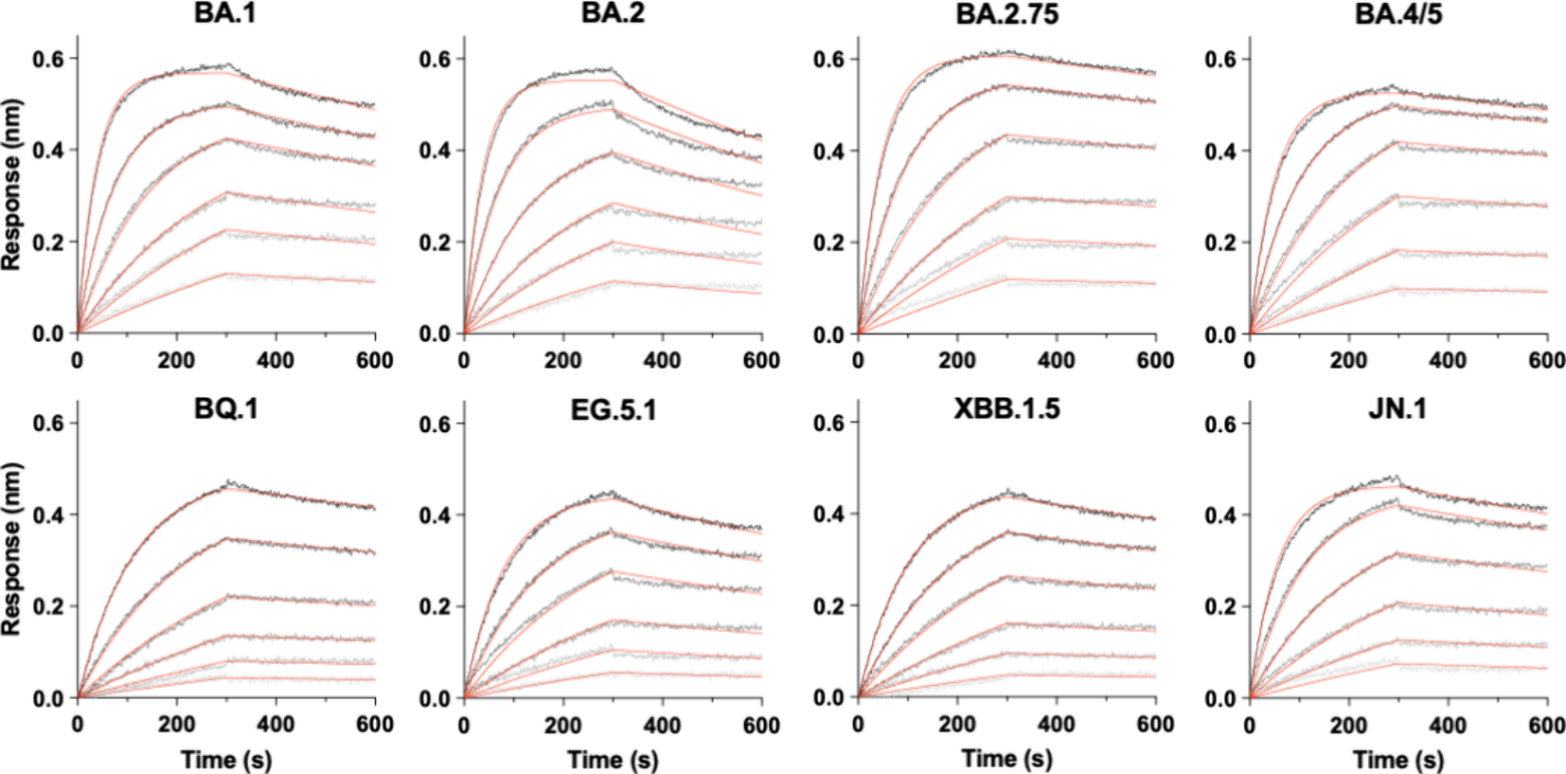
Biolayer Interferometry kinetic analysis of Omicron variant receptor binding. BLI sensorgrams measuring the association and dissociation between surface-immobilized dimeric hACE2-Fc and Omicron S variants. The identities of the individual S variants are indicated above their corresponding panels. The BLI measurements were carried out using a range of S protein concentrations by two-fold serial dilutions from 50 nM to 1.563 nM shown in descending orders from dark to light gray, superimposed with the global fitting results in red lines.

Since the upward open conformation of the RBD is the prerequisite of ACE2 binding, we asked whether the reported cryo-EM structures of different Omicron S variants exhibit any intrinsic propensity to adopt the upward open conformation for the three RBDs that can be correlated with our MP findings. We hypothesize that ACE2 binding may follow a conformation selection process, in which the pre-existing population of RBDs in the receptor-accessible ″up″ conformation would favor ACE2 binding through multivalency. Additionally, S variants that exhibit more S:ACE2_2_ and S:ACE2_3_ populations in the MP analysis may also exhibit more 2 RBD-up and 3 RBD-up conformations readily available for ACE2 binding. To test our hypothesis, we analyzed the distribution of RBD tilting angles from all reported cryo-EM structures of SARS-CoV-2 S variants in their apo forms, ACE2-bound forms, and antibody-bound forms. As of May 2025, 62, 33, and 88 structures of the apo forms, ACE2-bound forms, and antibody-bound forms, respectively. We calculated the angle θ between a vector from the RBD’s center of mass (COM) to its hinge residue C α atom (residue 330) and the plane formed by the Cα atoms of the three hinge residues of the trimeric assembly (Figure 5A). An angle θ < 50° was defined as a “down” (receptor-inaccessible) conformation, and an angle θ > 50° as an “open” (receptor-accessible) conformation.

**Figure 5:**
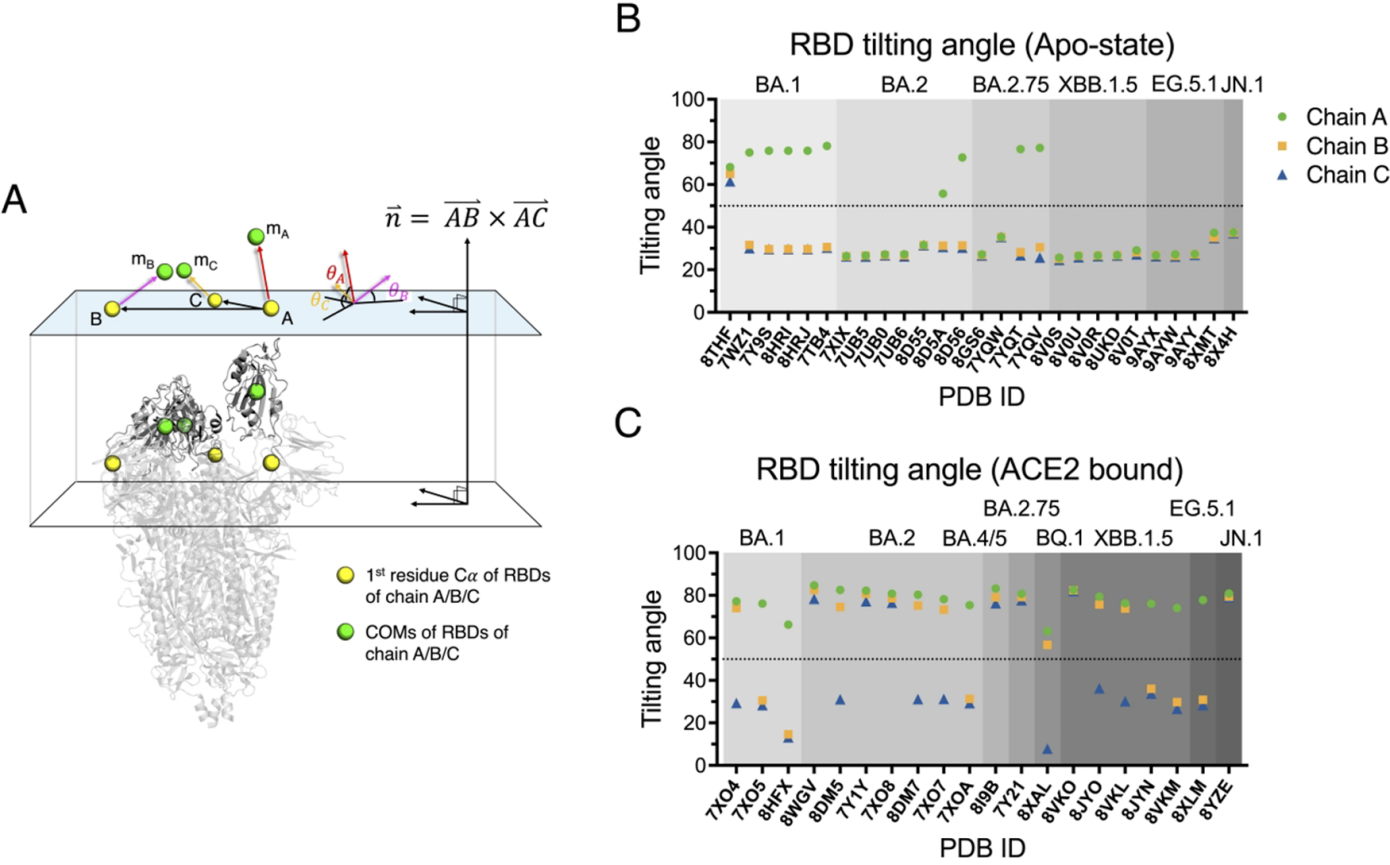
RBD tilting angle statistics of SARS-CoV-2 S structures reported in PDB. (A) Schematic definition of the tilting angle θ. The plane is defined by the three hinge C α atoms (A, B, and C). Its normal vector is calculated as the cross product of vectors AB and AC. The tilting angle θ is then determined as the angle between this plane and the vector from the hinge Cα atoms to the RBD’s center of mass (COM, m_A_, m_B_, and m_C_). (B) RBD tilting angle analysis of Apo-form S protein cryo-EM structure. (C) RBD tilting angle analysis of ACE2-bound S protein structure. Each dot represents a single RBD in a trimer, color-coded by protomer: green circles (chain A), orange squares (chain B), and blue triangles (chain C). Variants are arranged left to right in evolutionary order, with background gray scale progressing from light (early) to dark (late). The dashed line in panels A and B indicates a 50° tilting angle, used as a reference threshold to determine the RBD’s up/down conformation.

Consistent across all reported SAR-CoV-2 S protein structures, ACE2-bound forms and antibody-bound forms showed at least one, and often two or three RBD-up conformation (θ > 50°). In contrast, most apo-form RBDs, particularly for the Omicron subvariants, adopted an RBD-down conformation (θ < 50°; Figure S5). This prevalence of down conformations in apo forms may partly reflect the stabilizing effect of HexaPro modifications.^11^ Even with increased viral infectivity and disease severity over SARS-CoV-2 evolution, no clear correlation was found with “up/down” RBD conformational distributions. This broad conformational diversity across all variants is consistent with both our PDB analysis and prior cryo-EM reports.^23^

We further focused on the RBD-up/down populations derived from the cryo-EM structures of apo Omicron S variants, all of which harbor the HexaPro substitution. Of the limited number of cryo-EM structures (n = 27 for BA.1, BA.2, BA.2.75, XBB.1.5, EG.5.1, and JN.1), only one structure (PDB ID 8THF, corresponding to BA.1) exhibits a 3 RBD-up conformation. The remaining five BA1 S protein structures all show a 1 RBD-up conformation. Two BA.2 and two BA.2.75 structures show an 1 RBD-up conformation, whereas all the other structures adopt the all RBD down conformations (Figure 5B). Comparing these structures with our MP data, we found no correlation between the intrinsic propensity to adopt the RBD-up conformation in the cryo-EM structures and our MP results.

In the ACE2-bound structures (n = 20), fully ACE2-bound structures (S:ACE2_3_) are reported for BA.2, BA.4/5, BA.2.75, XBB.1.5, and JN.1 (Figure 5C). Nonetheless, we did not find an obvious correlation between the cryo-EM structures and our MP findings. We, therefore, concluded that the static, prebinding conformation of the S protein trimer deduced by cryo-EM single particle analysis may not reflect the true binding events in terms of the binding stoichiometry in solution. The use of single-molecule MP analysis in this study has enabled direct quantification of ACE2 binding stoichiometries for a panel of key SARS-CoV-2 Omicron subvariants. The primary finding is that the evolution of receptor engagement is not monotonous in the context of the relative populations of different binding stoichiometries.

## Discussion

Our MP data revealed an initial upward trajectory toward increased multivalency, characterized by higher populations of S:ACE2_2_ and S:ACE2_3_ complexes, which could facilitate host attachment, as exemplified by the BA.4/5 and BA.2.75 S variants. This trend was followed by a clear reversal in later lineages, culminating in significantly attenuated ACE2 engagement in EG.5.1 and JN.1. Such a reversal represents a counterintuitive evolutionary outcome. A functional trade-off between receptor binding and immune evasion may be associated with the changes, but further clinical validations are necessary to establish the causal relationship. It is also possible that the differences in ACE2 binding stoichiometry is due to shifting RBD up/down transition kinetics, RBD rebinding dynamics, or cooperative effects that cannot be captured by equilibrium MP. Nonetheless, there exists a correlation between the increasing population of unbound S proteins (from 38% in BA.1 to 58.1% in JN.1) and the accumulation of S protein mutations (from 37 to 66; Figure 4). Notably, the JN.1 variant, which exhibits the least ACE2-bound population among the Omicron variants according to our MP analyses herein, harbors two unique N-linked glycosylation sites at N245 and N354. These structural additions, visualized in our GlycoSHIELD models,^12^ are positioned to sterically impede ACE2 access (Figure 4B), suggesting that enhanced antibody evasion may outweigh the cost of reduced ACE2 binding avidity in a host population with substantial pre-existing immunity. Nonetheless, the changes in ACE2 binding stoichiometry do not reflect in the kinetic binding analysis by BLI, which shows fluctuations in the *K*_D_ values (Figure 4 and Table 2) that do not correlate with the MP measurements.

**Table 2:** Kinetics Parameters of Omicron S Variants Binding to ACE2 Derived from BLI Measurements^a^

	BA.1	BA.2	BA.2.75	BA.4/5	BQ.1	XBB.1.5	EG.5.1	JN.1
*K*_D_ (nM)	0.98	1.72	0.59	0.63	1.78	2.03	2.67	1.31
*k*_*on*_ (M^–1^ s^–1^)	5.2× 10^5^	5.3 × 10^5^	4.2 × 10^5^	4.0 × 10^5^	1.7 × 10^5^	1.9 × 10^4^	2.4 × 10^4^	3.5 × 10^5^
*k*_*off*_ (s^–1^)	5.1 × 10^–4^	9.1 × 10^–4^	2.5 × 10^–4^	2.5 × 10^–4^	3.1 × 10^–4^	3.9 × 10^–4^	6.5 × 10^–4^	4.6 × 10^–4^

a The association rate constant (*k*_on_), dissociation rate constant (*k*_off_), and apparent dissociation constant (*K*_D_) were derived through globally fitting of the sensorgrams to a 1:1 binding model.

Our stoichiometric analysis further resolves “enhanced binding” into distinct mechanistic strategies. The BA.4/5 variant preferentially populates multivalent binding states (24% S:ACE2_2_ and 10% S:ACE2_3_), consistent with a conformationally permissive architecture that supports higher-order engagement. In contrast, BA.2.75 minimizes its apo-state population (20%) by maximizing formation of the initial 1:1 S:ACE2 complex (62%), indicative of optimization for high-avidity primary binding rather than multivalency. Across all variants, the consistently low occupancy of the fully saturated S:ACE2_3_ state (<11%) supports models invoking steric hindrance or negative cooperativity between protomers during sequential ACE2 binding.

Our MP-derived patterns correlate with known shifts in infectivity and tropism across Omicron sublineages: the high-avidity recruitment signature of BA.4/5 aligns with its rapid entry kinetics, whereas the attenuated binding occupancy of JN.1 reflects an evolutionary shift where the virus prioritizes immune concealment over receptor-binding capacity.^24^ This suggests that while the environment may define the baseline occupancy, the sequence-specific differences in RBD accessibility captured by our MP platform represent the fundamental biophysical changes driving the continued evolution of SARS-CoV-2. Nonetheless, these changes in ACE2 binding stoichiometry are not reflected in the kinetic BLI measurements when the dimeric hACE2-Fc construct is immobilized on the BLI sensor tip to measure the on- and off-rates of S protein binding (Figure 4 and Table 2). Cryo-EM single particle analysis (SPA) prioritizes high-resolution particle imaging to focus on convergent image superposition, such that the minor populations are often excluded from further analyses. Few studies have systematically quantified the relative populations of the RBD of SARS-CoV-2 S variants in the up and down conformations^23^ or in other CoV systems.^25^ MP analysis, therefore, offers an orthogonal solution to quantitatively investigate the dynamic and complex protein–protein interactions under physiologically relevant conditions in vitro.

Avidity plays a key role in multivalent binding systems, such as trimeric S proteins compared to dimeric ACE2. Depending on the construct used—isolated RBD, monomeric ACE2 ectodomain, trimeric S proteins, or dimeric hACE2-Fc fusion—apparent binding affinities measured by BLI can differ by up to 2 orders of magnitude.^26^ SARS-CoV-2 virions typically display 25–40 S trimers,^27^ but only a few (2–5) engage the host cell during initial attachment.^28^ Cryo-EM SPA has revealed the structure of two trimeric S proteins from human CoV-229E bound to dimeric human aminopeptidase N (hAPN), illustrating receptor engagement when anchored on membrane environments.^29^

In our current experimental design, the intrinsic stoichiometries measured by MP provide a mechanistic basis for enhanced attachment avidity. Even if steric constraints limit the number of S that can simultaneously reach the cell surface, the ability of a single trimer to capture multiple ACE2 molecules—facilitated by ACE2 clustering in lipid rafts and the high flexibility of the S stalk—significantly increases the probability of stable tethering.^30^ We acknowledge that stoichiometric distributions can change when S proteins are in native lipid environments, where both S proteins and ACE2 move laterally on viral and host membranes rather than floating in solution. The gap between physiological conditions and in vitro measurements is a fundamental limitation of our experiments. Furthermore, all MP and BLI measurements were conducted using the HexaPro-stabilized versions of the S variants,^19^ as this approach enables the production of mg-scale recombinant S proteins in a cost-effective manner. In our experience, utilizing the 2P version substantially decreased production yields (Figure S6).

Previous studies employing MP measurements and mass spectrometry-based glycoproteomic analyses have indicated that the HexaPro modification promotes the RBD in a downward, closed conformation.^11^ Although this limitation exists, most cryo-EM Omicron S protein structures in the PDB contain the HexaPro modification (Figure 5). Artificially stabilizing the closed state of these S proteins is necessary for systematic, comparative, and quantitative analyses using a consistent baseline. However, it is crucial to consider this factor when translating our in vitro findings into recommendations for real-world clinical scenarios.

## Conclusions

This study, conducted with the HexaPro-stabilized soluble ectodomains, providing an in vitro and biophysical view for the potential evolutionary trends. Our results demonstrate that ACE2 binding stoichiometry is a key, dynamically regulated parameter rather than a fixed consequence of binding affinity alone. By providing direct, solution-based measurements of stoichiometric populations, we shift the perspective from a purely affinity-centric view toward a model in which receptor engagement reflects a balance between multivalency, conformational dynamics, and immune evasion.

The observed stoichiometric diversity highlights potential evolutionary strategies and suggests that differential receptor engagement may inform immunogen design approaches aimed at stabilizing vulnerable conformations or limiting multivalent binding. More broadly, the MP-based framework presented here should be broadly applicable to characterizing receptor engagement heterogeneity in other emerging viral pathogens and engineered vaccine antigens. Future studies integrating these quantitative stoichiometric signatures with cell-based entry assays and targeted mutational analyses, including validation of the N245 and N354 glycosylation sites, will be critical for linking biophysical behavior to functional outcomes.

## Supplementary Material



## Notes

Characterization and quality control of purified SARS-CoV-2 S ectodomains SDS–PAGE (Figure S1) and MP (Figure S2); stoichiometric distribution across different mixing ratios (Figure S3); reproducibility of ACE2 binding stoichiometry analyses across Omicron S variants determined by MP (Figure S4); RBD tilting angle analysis and conformational distributions derived from SARS-CoV-2 S protein cryo-EM structures (Figure S5); comparative purification of Omicron S protein constructs (Figure S6) (PDF)
