# Supplementary material for: Mass Photometry Reveals Distinct ACE2 Binding Stoichiometries across SARS-CoV‑2 Omicron Subvariants
Source: J Phys Chem B. 2026 Apr 30;130(19):4961–9. doi: 10.1021/acs.jpcb.6c00027 (PMC13181760; doi:10.1021/acs.jpcb.6c00027)
Supplement: Supplementary file 1 [file jp6c00027_si_001.pdf]

Mass Photometry Reveals Distinct ACE2 Binding Stoichiometries Across SARS-CoV-2 Omicron Subvariants

Wei-Cheng Hsiao[1,2†], Tsung-Sheng Chiang[1,2†], Yu-Xi Tsai[1], Min-Feng Hsu[1], Shang-Te Danny Hsu[1,2,3,4,*]

1. Institute of Biological Chemistry, Academia Sinica, Taipei 11529, Taiwan
2. Institute of Biochemical Sciences, National Taiwan University, Taipei 10617, Taiwan
3. International Institute for Sustainability with Knotted Chiral Meta Matter (WPI-SKCM^2), Hiroshima University, 1-3-1 Kagamiyama, Higashi-Hiroshima, Hiroshima 739-8531, Japan
4. Department of Biochemistry, Microbiology, and Immunology, Faculty of Medicine, University of Ottawa, Ottawa, Ontario K1H 8M5, Canada

[†] These authors contributed equally to this work

* Corresponding author: sthsu@as.edu.tw

This file contains

Figures S1-6

Tables S1

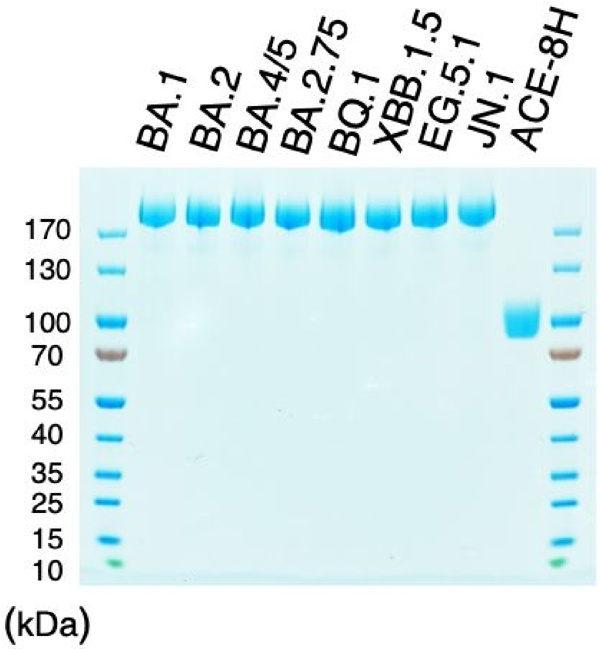

Supplementary Figure S1. Quality control of purified SARS-CoV-2 S proteins and ACE2. SDS-PAGE image of Omicron subvariants (BA.1, BA.4/5, BA.2.75, BQ.1, XBB.1.5, EG.5.1, and JN.1) and human ACE2, confirming the purity of the recombinant proteins.

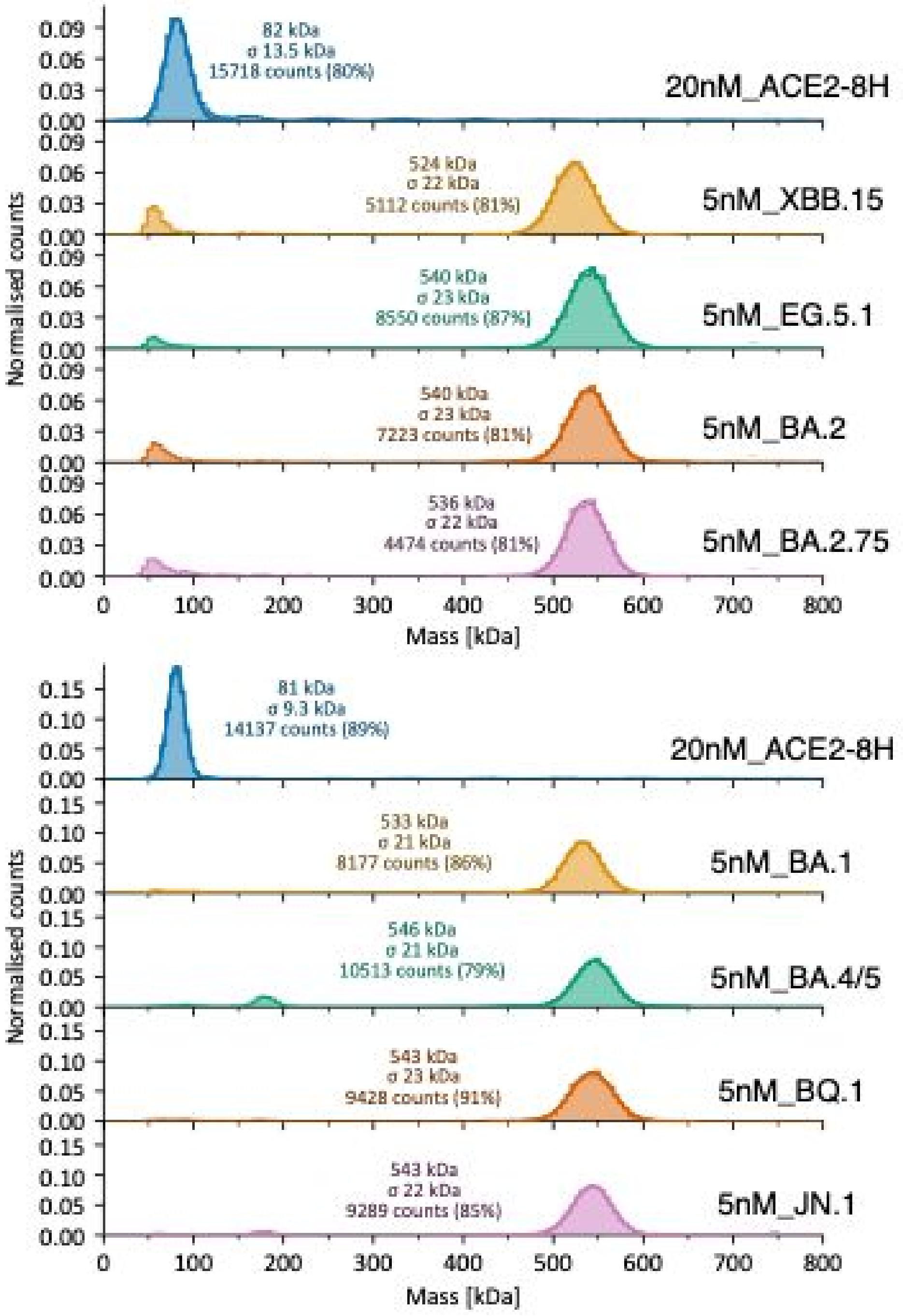

Supplementary Figure S2. MP histograms of recombinant S variants and ACE2 in isolation. The protein concentrations and their identities are indicated on the right.

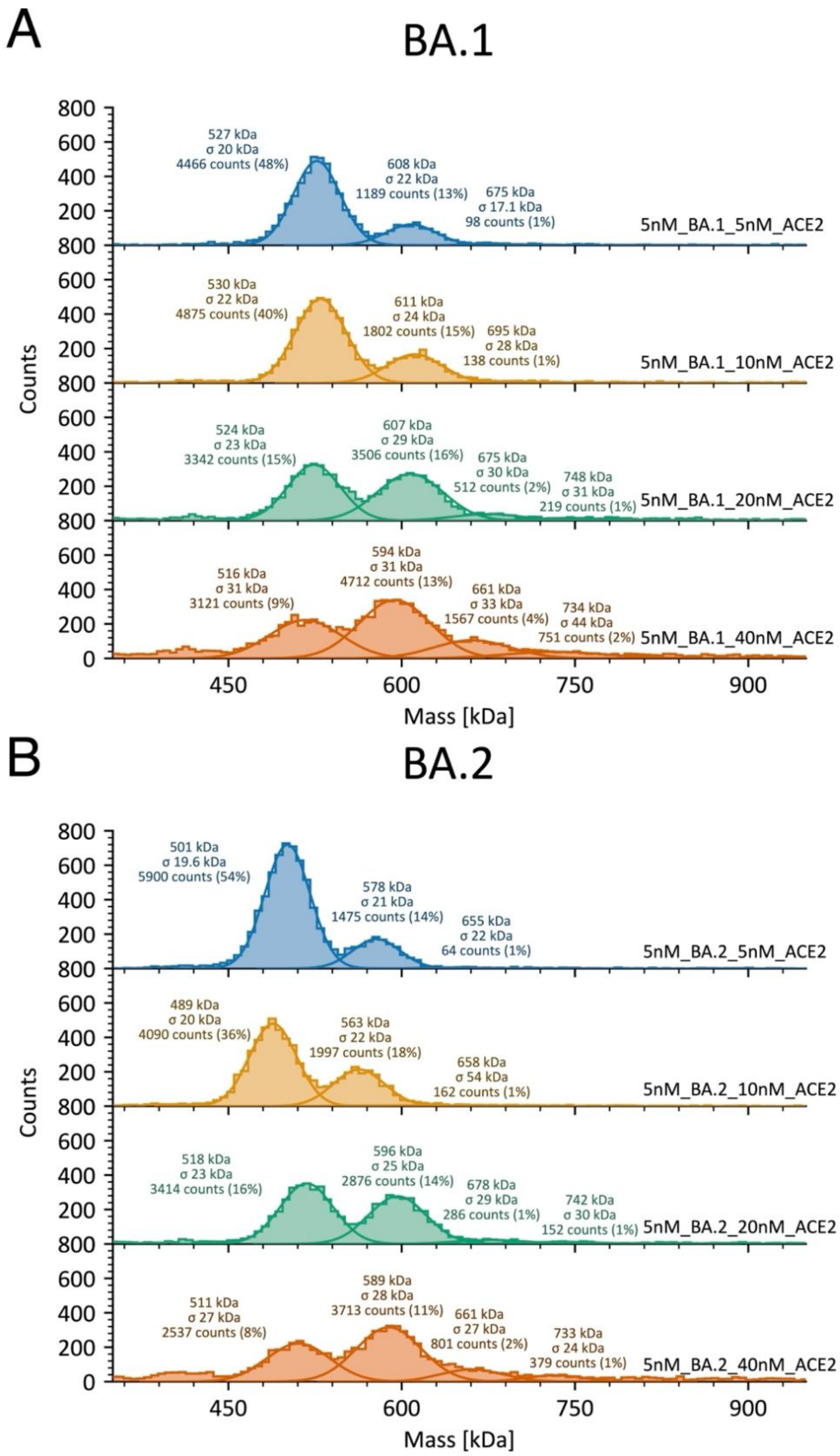

Supplementary Figure S3. MP histograms of S variants binding to ACE2 in different pre-mixing ratios. The histograms are plotted in descending order for 1:1, 1:2, 1:4 and 1:8 mixing ratios for BA.1 (A) and BA.2 (B). The populations of the individual binding stoichiometries are fitted to Gussian distributions with the relative populations indicated above individual peaks.

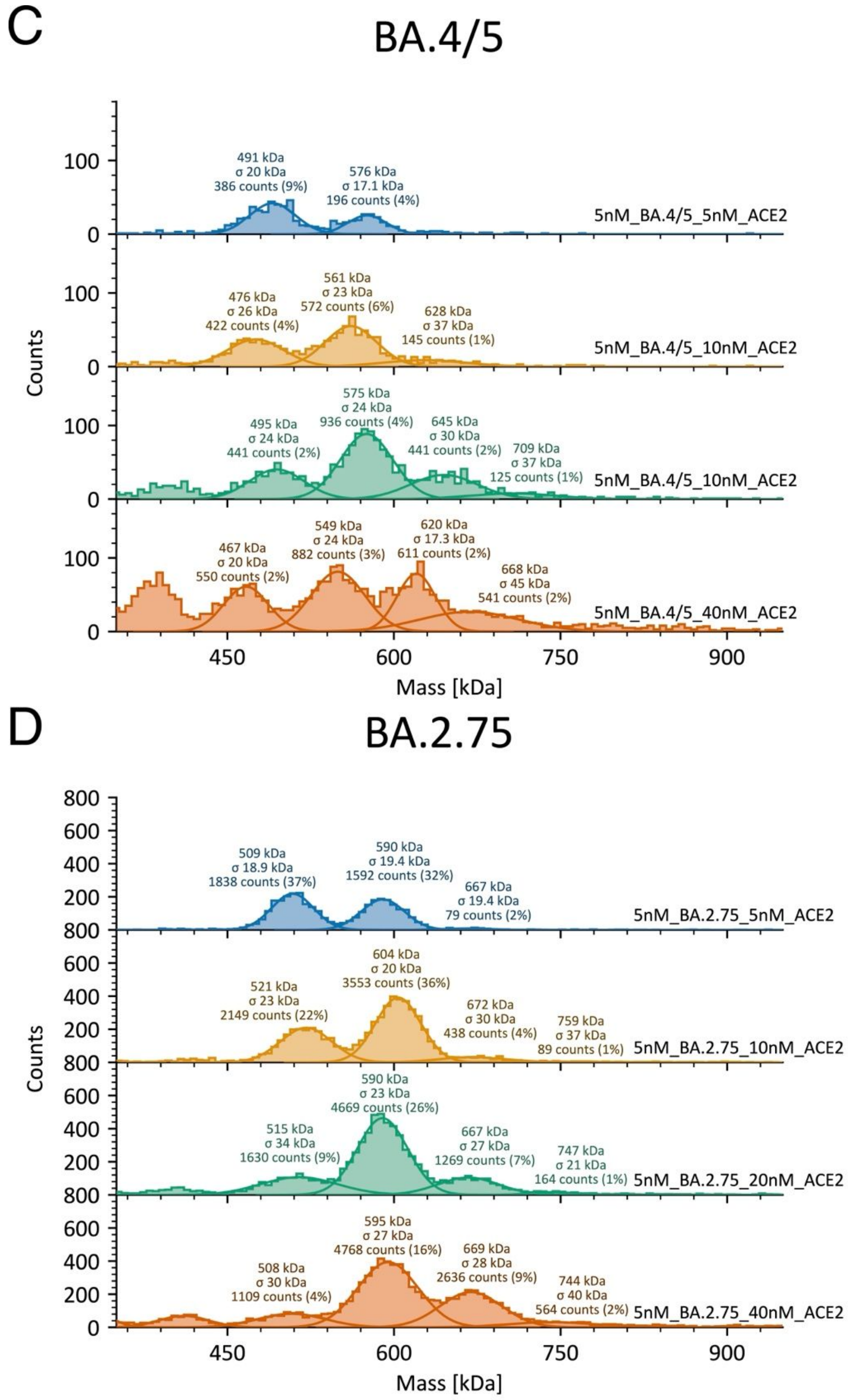

Supplementary Figure S3 (continued). MP histograms of S variants binding to ACE2 in different pre-mixing ratios. The histograms are plotted in descending order for 1:1, 1:2, 1:4 and 1:8 mixing ratios for BA.4/5 (C) and BA.2.75 (D). The populations of the individual binding stoichiometries are fitted to Gussian distributions with the relative populations indicated above individual peaks.

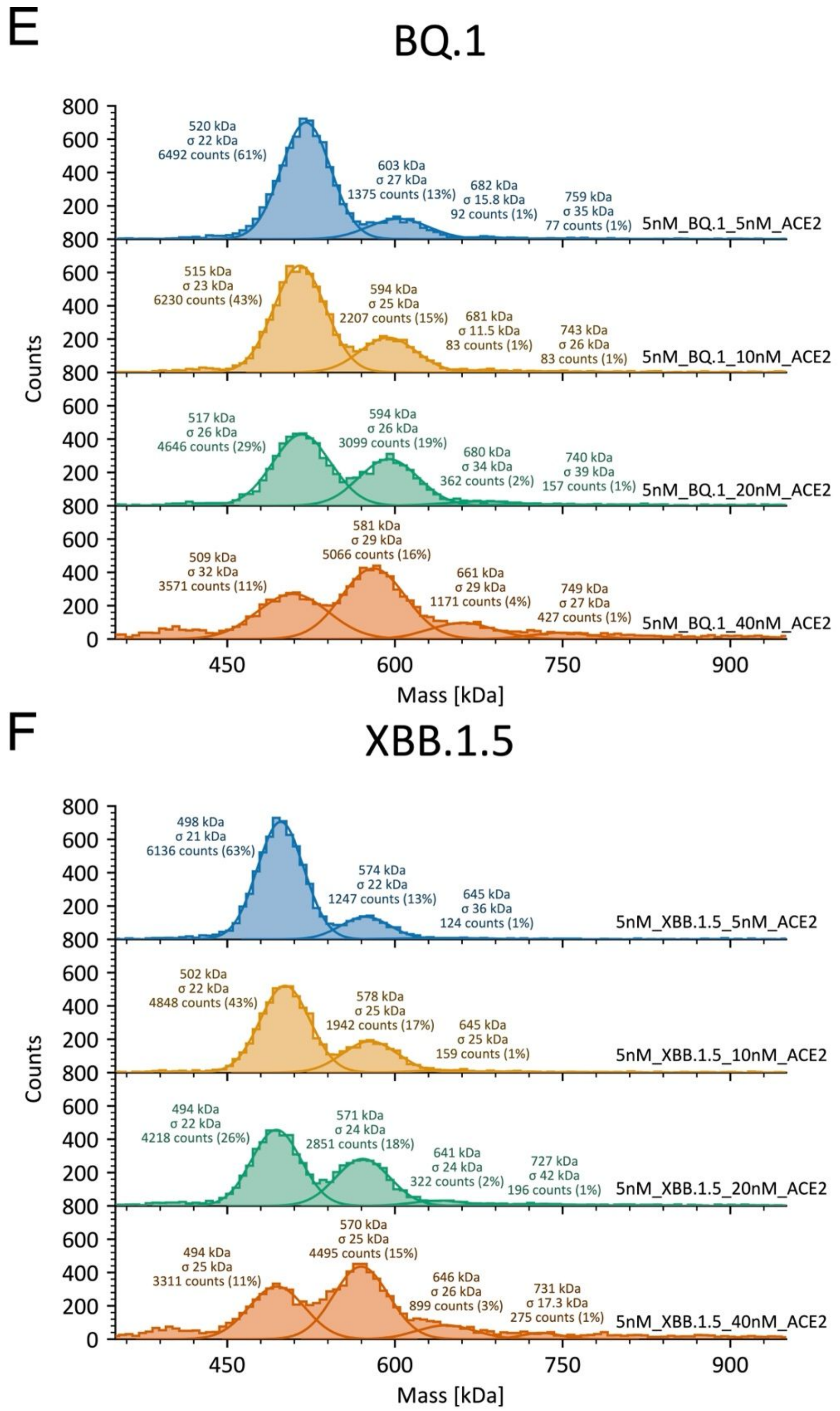

Supplementary Figure S3 (continued). MP histograms of S variants binding to ACE2 in different pre-mixing ratios. The histograms are plotted in descending order for 1:1, 1:2, 1:4 and 1:8 mixing ratios for BQ.1 (E) and XBB.1.5 (F). The populations of the individual binding stoichiometries are fitted to Gussian distributions with the relative populations indicated above individual peaks)

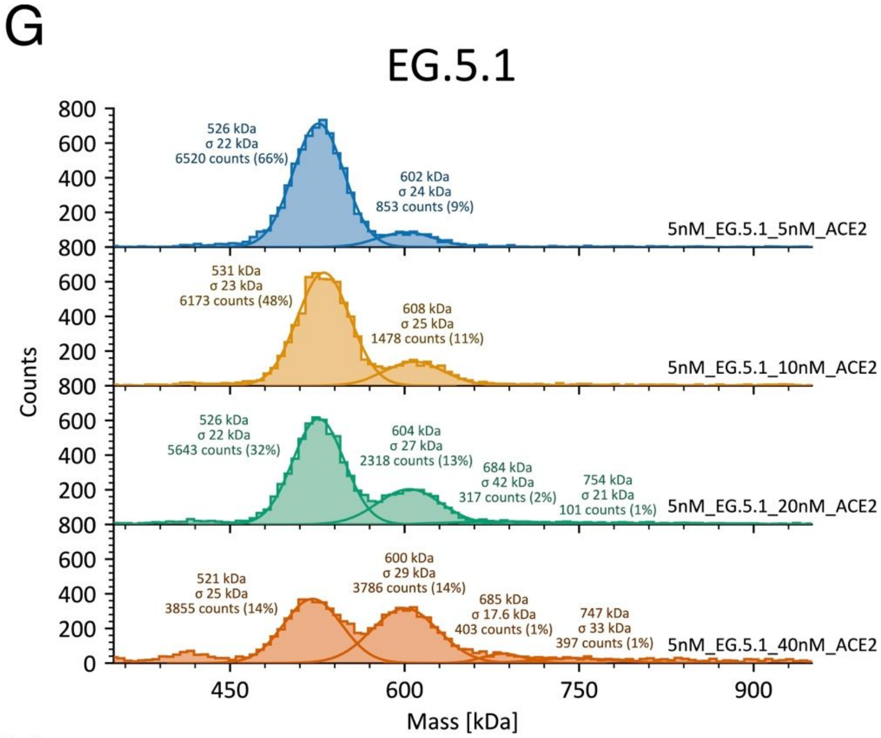

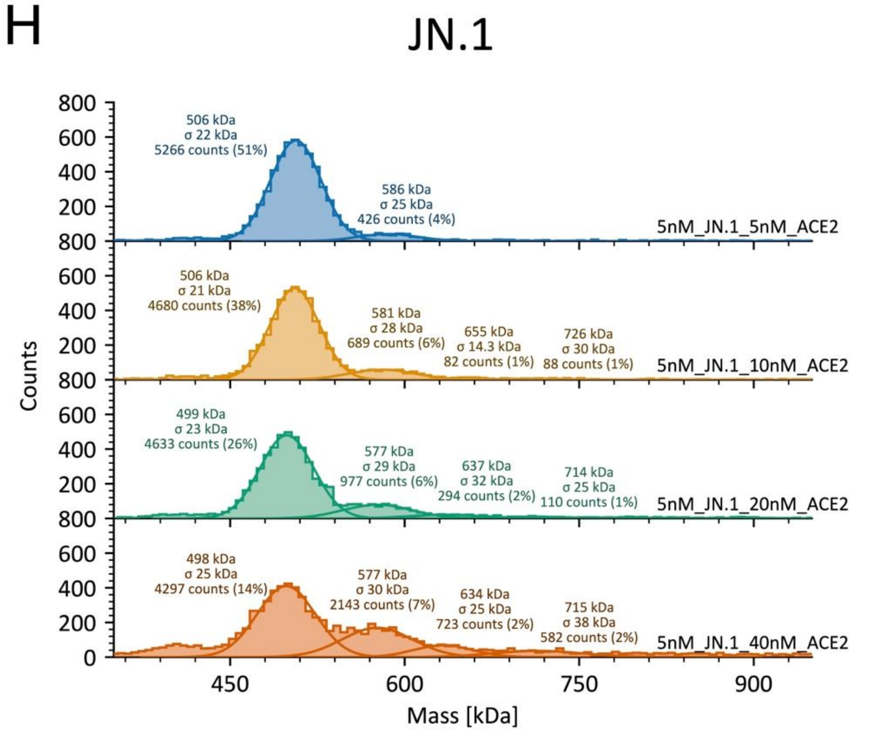

Supplementary Figure S3 (continued). MP histograms of S variants binding to ACE2 in different pre-mixing ratios. The histograms are plotted in descending order for 1:1, 1:2, 1:4 and 1:8 mixing ratios for EG.1 (G) and JN.1 (H). The populations of the individual binding stoichiometries are fitted to Gussian distributions with the relative populations indicated above individual peaks

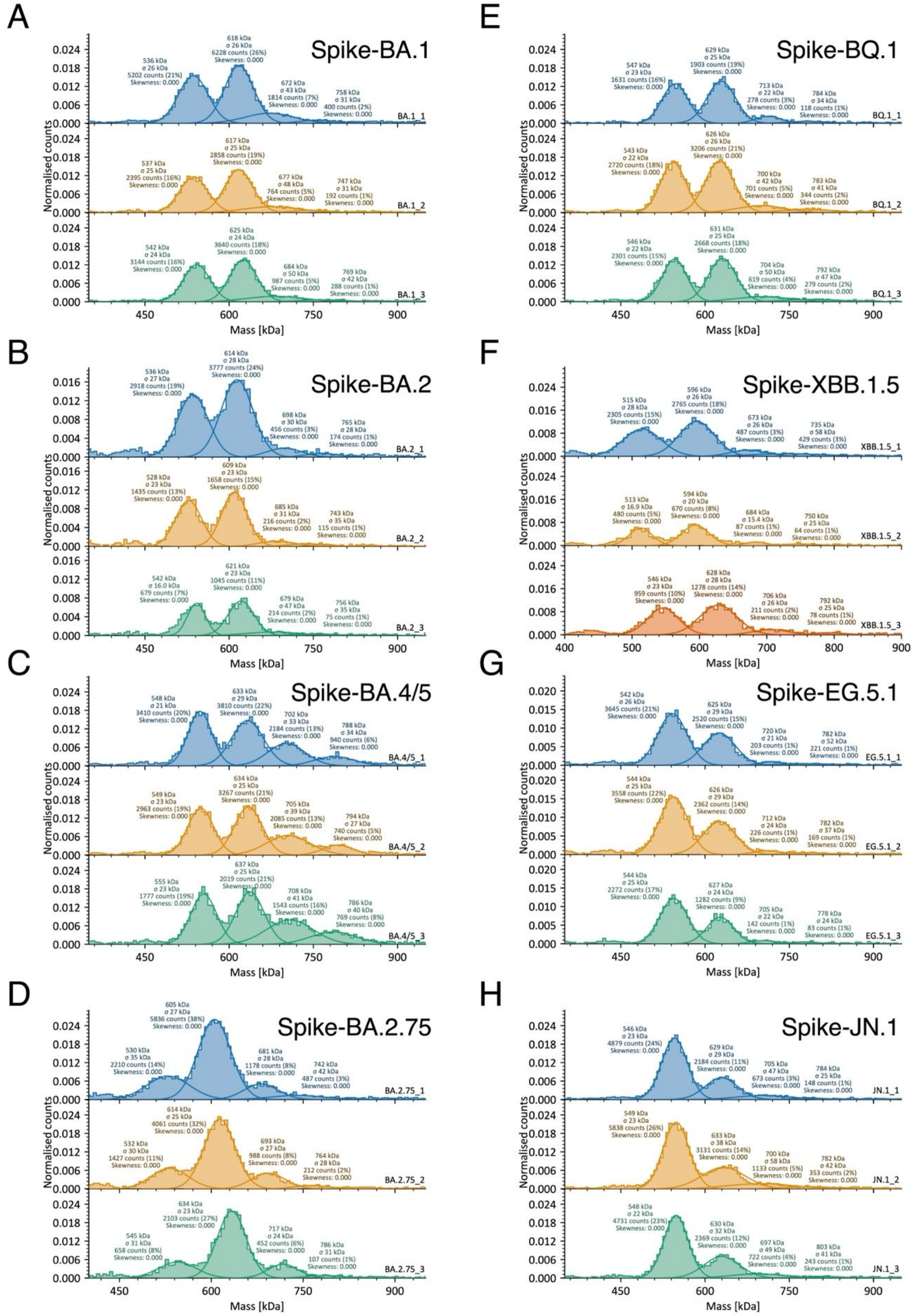

Supplementary Figure S4. MP histograms of S variants binding to ACE2 in a 1:4 pre-mixing ratio in technical triplicates.

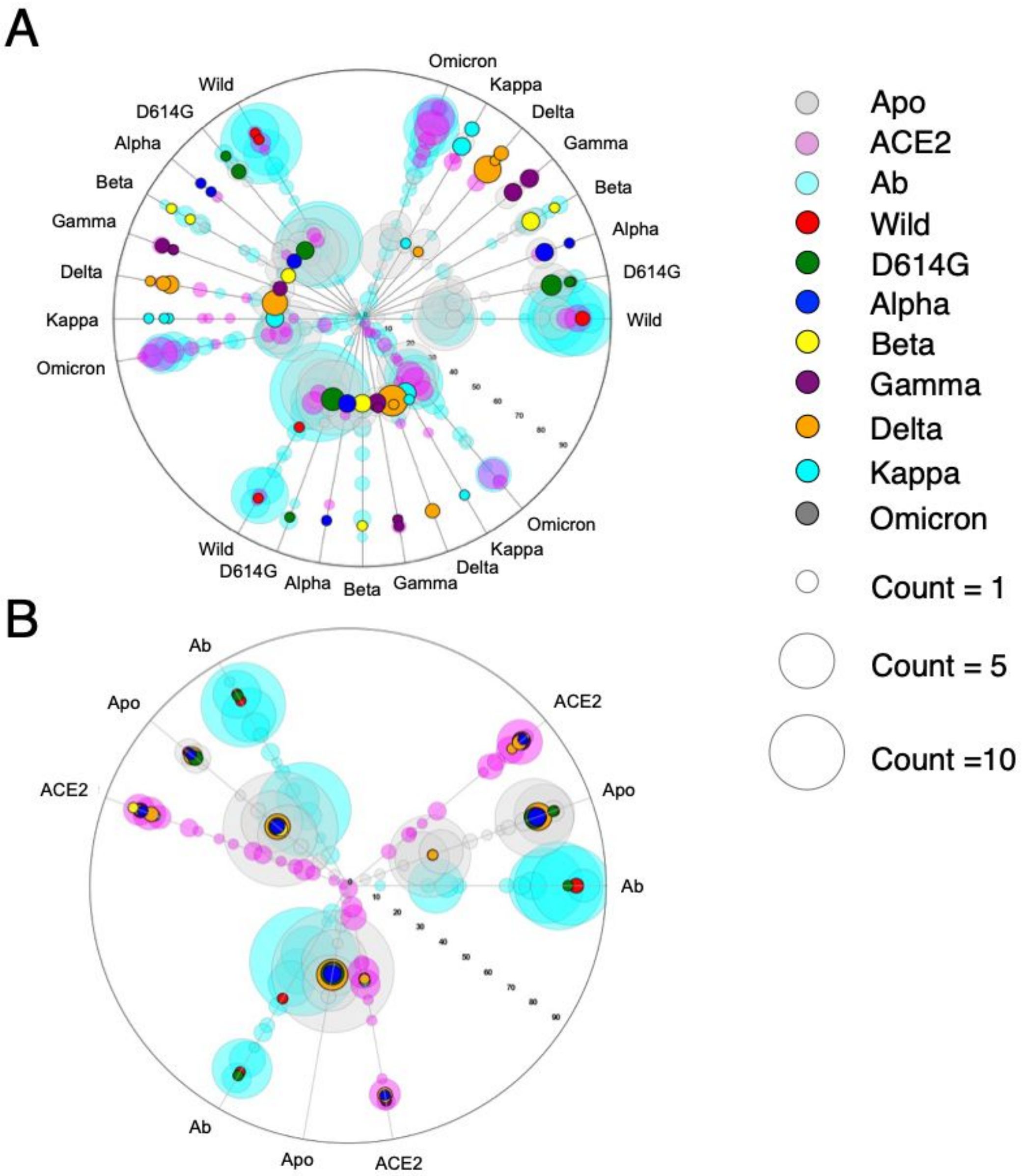

Supplementary Figure S5. RBD tilting angle analysis of SARS-CoV-2 S protein structures reported in the PDB. Radar plots illustrate RBD up/down conformation distributions across SARS-CoV-2 variants (defined by θ > 50˚ for "up", θ < 50˚ for "down"; see Fig. 3C). Each plot has three segments for trimeric chains (top right: A, top left: B, bottom: C). Circles are colored by binding state (gray: Apo, magenta: ACE2-bound, cyan: Antibody-bound). Circle radius reflects the count of structures, and angle (inner to outer) corresponds to the tilting angle θ.

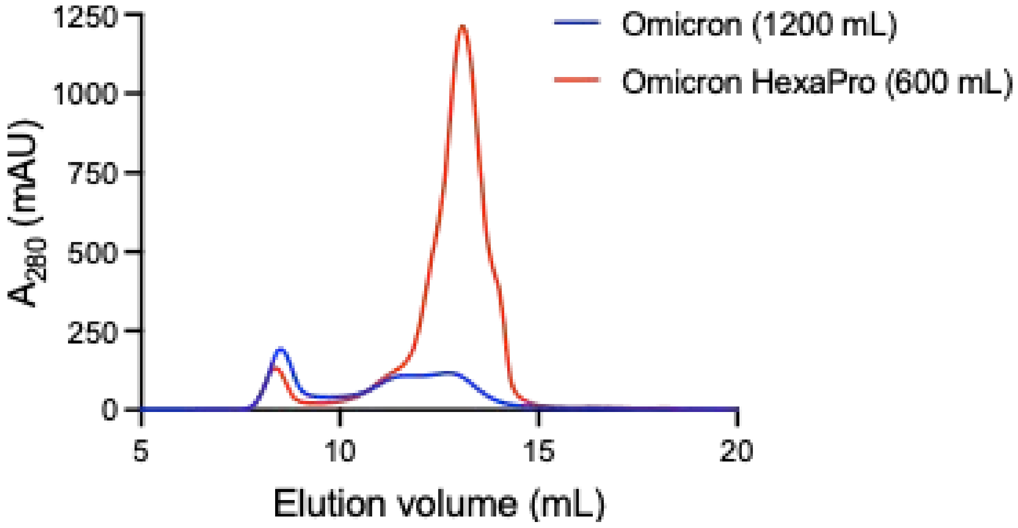

Supplementary Figure S6. Comparison of recombinant S proteins of BA.1 with and without HexaPro. Representative SEC chromatograms of BA.1 S protein harboring 2P (blue) and HexaPro (red) modifications. The yield of the HexaPro variant is approximately 20-fold higher than that of the 2P variants considering the difference in the culture size (600 mL for HexaPro and 1200 mL for 2P).

Supplementary Table S1. Details of glycoforms and glycosylation sites used for GlycoSHIELD modeling of the S protein of JN.1.

Glycan Code	Composition (Hex:HexNAc:Fuc:NeuAc)	Type/classification	N-glycosylation sites
2500	2:5:0:0	Truncated Complex	62, 121, 242, 599, 705, 713, 797, 1070
2800	2:8:0:0	Extended Core	231
3610	3:6:1:0	Fucosylated Complex	653
4310	4:3:1:0	Mono-sialylated Complex	147, 328, 340, 351, 612, 1130
4410	4:4:1:0	Bi-antennary Complex	1094
4511	4:5:1:1	Sialylated Complex	163
5310	5:3:1:0	Hybrid / Complex	279, 1173
6511	6:5:1:1	Sialylated Tri-antennary	73, 1194
4400	4:4:0:0	Complex (No Fuc)	1158
